# Promoter Engineering of the Surfactin Operon Enhances Surfactin Production in the Environmental Strain *Bacillus subtilis* RI4914

**DOI:** 10.1007/s00284-026-05037-3

**Published:** 2026-06-30

**Authors:** Michelle Fernandes Almeida, Fernanda de Souza Freitas, Bruna Almeida Leão Ayupe, Janine T. Bossé, Denise Mara Soares Bazzolli, Victor Satler Pylro, Pedro Marcus Pereira Vidigal, Humberto Josué de Oliveira Ramos, Edmo Montes Rodrigues, Marcos Rogério Tótola

**Affiliations:** 1 Department of Microbiology, Federal University of Viçosa, Viçosa, Minas Gerais Brazil; 2 Department of Infectious Disease, Imperial College London, London, UK; 3 Department of Biology, Federal University of Lavras - UFLA, Lavras, Minas Gerais Brazil; 4 Biomolecule Analysis Center (NuBioMol), Federal University of Viçosa, Viçosa, Minas Gerais Brazil; 5 Department of Biochemistry and Molecular Biology, Federal University of Viçosa, Viçosa, Minas Gerais Brazil; 6 Department of Education, Federal Institute of Education, Science and Technology of Ceará - campus Camocim, Camocim, Ceará Brazil

## Abstract

This study aimed to enhance surfactin production in *Bacillus subtilis* by replacing the native promoter of the surfactin synthase operon (P*srfA*) with the strong IPTG-inducible promoter Pgrac to address challenges in large-scale biosurfactant production. The engineered strain was developed through homologous recombination, creating an integration cassette fused via overlap extension PCR and transformed into *B. subtilis* RI4914. Surfactin yields were assessed via oil spreading and UPLC-MS. Results showed that surfactin synthesis in the modified strain was nearly absent without IPTG. With IPTG, however, production surged 8.4-fold compared to the wild type, reaching 4.8 g/L. Productivity and carbon source yield also improved 8-fold and 11-fold, respectively. This promoter engineering strategy significantly enhanced surfactin production, with the observed increase being consistent with the expected behavior of IPTG-inducible Pgrac systems in Bacillus subtilis, suggesting an effective regulatory response associated with the tested expression system.

**Supplementary Information:**

The online version contains supplementary material available at 10.1007/s00284-026-05037-3.

## Introduction

Surfactin is a lipopeptide biosurfactant produced by bacteria belonging to the genus *Bacillus* [1]. It is synthesized non-ribosomally by the multi-modular enzyme complex surfactin synthetase, composed of three large non-ribosomal peptide synthetases (SrfAA, SrfAB, and SrfAC). The genes that code for peptide synthetases are organized in the 27 kb *srfA* operon, which is controlled by the promoter P*srfA* [2, 3].

With its antibacterial, antiviral, antifungal, antibiofilm, anticancer, and anti-inflammatory properties, surfactin is regarded as one of the most promising biosurfactants and is highly valued by the food, pharmaceutical, and agricultural sectors [4]. This molecule is ideal for environmental applications such as bioremediation and oil recovery, as well as for the cleaning and cosmetics sectors, due to its remarkable surface activity and thermal and pH stability [5–8].

Despite its great potential, industrial applications of surfactin remain limited because large-scale production is constrained by high production costs and low yields in wild strains [1, 9, 10]. Consequently, enhancing surfactin production has been widely pursued by scientists and industries, including the optimization of both the fermentation process and bacterial strains [11–15]. However, the titers achieved with fermentation optimization are still limited for commercial applications. Therefore, developing genetically modified surfactin-overproducing strains is of great relevance (9).

Promoter engineering is a promising strategy to enhance the transcription of the *srfA* operon and improve surfactin productivity to meet the requirements of scaled-up manufacturing [14, 16, 17]. In this work, we aimed to enhance surfactin production in the environmental strain Bacillus subtilis RI4914 by replacing the native P*srfA* promoter with a strong IPTG-inducible promoter.

## Materials and Methods

### Microorganisms and Plasmids

*Bacillus subtilis* RI4914 was used as a host strain in this study. This wild surfactin producer [18, 19] belongs to the culture collection of the Laboratório de Biotecnologia e Biodiversidade para o Meio Ambiente (LBBMA), Departamento de Microbiologia, Universidade Federal de Viçosa (UFV). It was isolated from the production water of the Rio-Itaúnas formation, Conceição da Barra, Espírito Santo, Brazil. *Escherichia coli* DH5α was used for plasmid construction and propagation.

The pHT43 plasmid was purchased from MoBiTec GmbH (Göttingen, Germany) and used as a template to amplify the P*grac* promoter, *lacI*, and *cat* genes to construct the integration cassette. pGEM-T Easy vector was used to clone the integration cassette. pT-*srfA* was constructed in this work and contains the integration cassette to transform *B. subtilis* to perform promoter switching.

### Construction of the Integration Cassette by Overlap Extension PCR

**Fig. 1:**
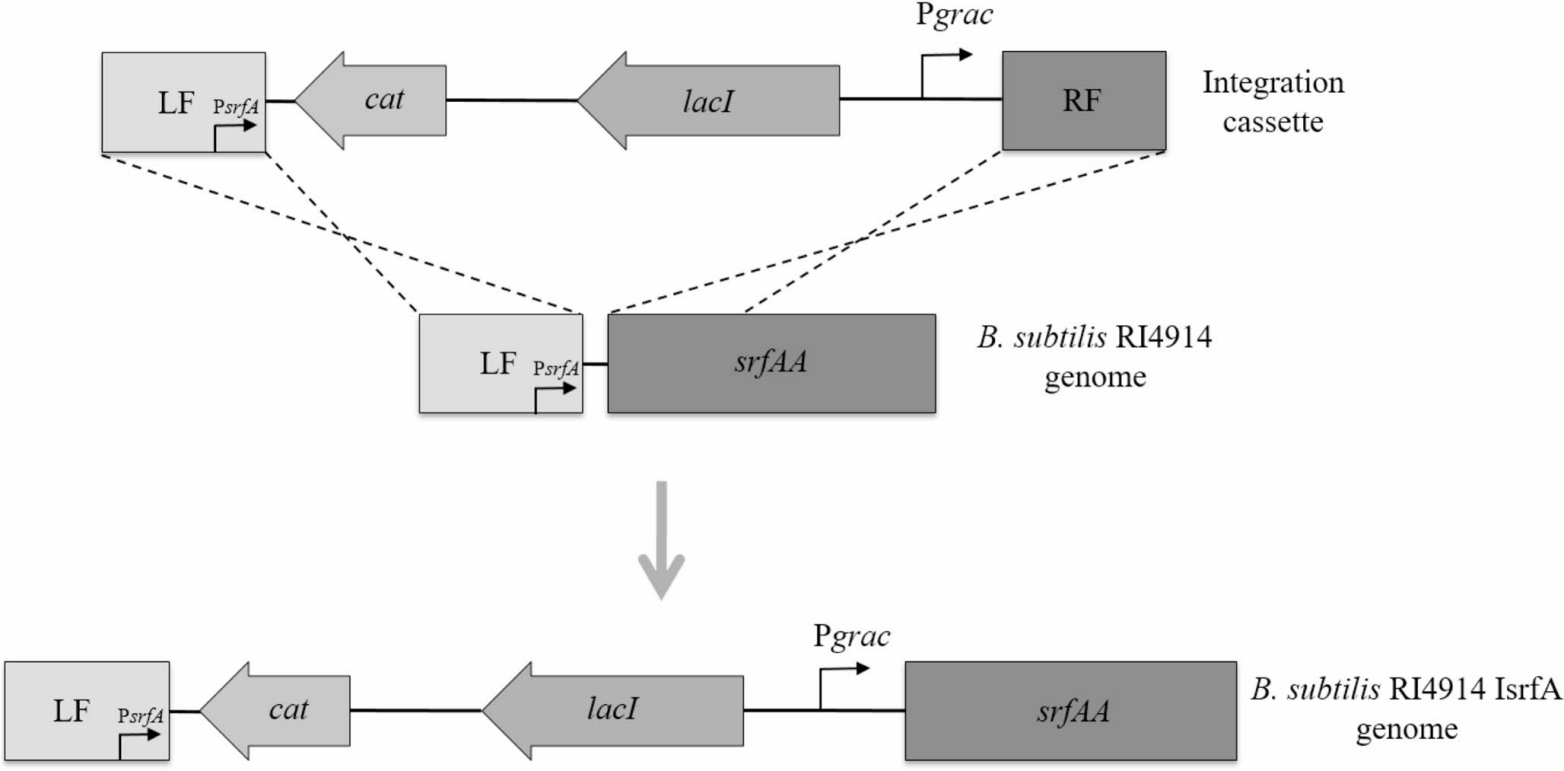
Schematic diagram of the strategy used in this work to exchange the P*srfA* promoter by double cross-over recombination. LF: Left flank, which includes part of the *hxlR* gene and the P*srfA* promoter; RF: Right flank, which includes part of the *srfAA* gene

To substitute the native chromosomal P*srfA* promoter with an inducible P*grac* promoter by double-cross homologous recombination, an integration cassette of DNA was constructed (Fig. 1). For this, three different DNA fragments were designed. *B. subtilis* RI4914 chromosomal DNA was isolated using the PowerSoil™ DNA Isolation Kit (MoBio, USA) and was used as the template for PCR amplification of the cassette left and right flank arms homologous to the regions upstream and downstream of the native promoter P*srfA*. The upstream flank (845 pb) contains the natural promoter P*srfA* and part of the gene hxlR. The downstream flank (773 pb) includes part of the first open reading frame of the *srfA* operon, srfAA. The third fragment of the cassette includes the new promoter region P*grac* (artificial promoter containing the strong promoter P*groE* fused to the IPTG-induced *lacO* operator), the *lacI* gene, and the chloramphenicol resistance gene (*cat*). This fragment was PCR amplified as a single fragment from the pHT43 vector.

**Fig. 2:**
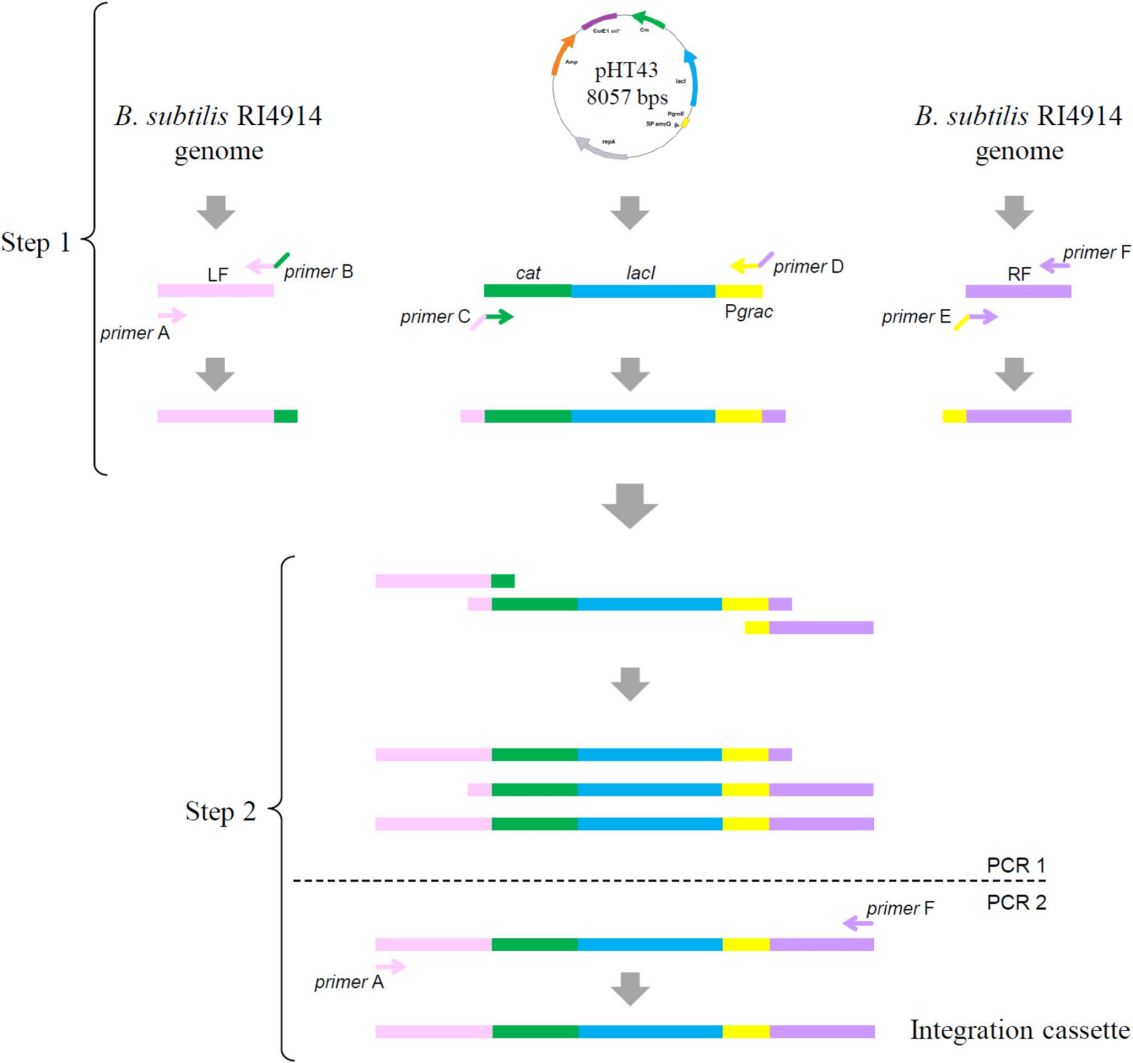
Construction of the integration cassette by Overlap Extension PCR. LF: Left flank; RF: Right flank

To construct the cassette, the three fragments were fused by overlap extension PCR (Fig. 2). In Step 1, each fragment was obtained separately by PCR. The primers (Table 1) were designed using the MacVector software [20]. PCR reactions were performed in a total volume of 50 µL (Table S1) at the following conditions: 2 min of denaturation at 94 °C, followed by 35 cycles of 15 s at 94 °C, 30 s at 57 °C (for left and right flank fragments) or 30 s at 54 °C (for P*grac* region), 1 min at 68 °C (for left and right flank fragments) or 3 min at 68 °C (for P*grac* region), and a final extension of 7 min at 68 °C. Amplicons were confirmed by agarose gel electrophoresis using 1 Kb and 100 bp DNA Ladder (Promega) as reference. PCR products free of non-specific bands were digested with the DpnI enzyme to remove template DNA remaining in the reaction mix. For reactions showing non-specific bands, it was not necessary to cleave with DpnI, since bands of a size compatible with that expected for the amplified DNA were purified by extraction of the band from the agarose gel. PCR products were purified using the illustraTM GFXTM PCR DNA and Gel Band Purification Kit (GE Healthcare), according to the manufacturer’s recommendations.

**Table 1:** List of primers used in this work

Primer	Identification	Sequence	Tm (°C)
Left Flank Forward	A	ACTTAGTCGACTGCAGTGATTGGCGGTAAATG	59.9
Left Flank Reverse	B	*GACTGTACTTTTTAC*GCAACGCATTTTCTCTTTCTTATCC	59.5
Chl-RBS Forward	C	*GAGAAAATGCGTTGC*GTAAAAAGTACAGTCGGCATTATCTC	58.4
Chl-RBS Reverse	D	*AAAAGTTATTTCCAT*TGATCCTTCCTCCTTTAATTGG	58.0
Right Flank Forward	E	*AAGGAGGAAGGATCA*ATGGAAATAACTTTTTACCCTTTAACGG	59.0
Right Flank Reverse	F	ACTTACTCGAGAGCAGCGATTGAAATACCGAAAG	59.9

The underline represents the overlapping tail added to the primers. The melting temperature (Tm) represented in the table corresponds to the Tm of the portion of the primers complementary to the fragment to be amplified, without the overlapping tail.

At Step 2, two consecutive PCR reactions were performed to obtain the integration cassette. The first reaction was carried out in a total volume of 25 µL, containing 1X of 10X High Fidelity Buffer, 3 mM of MgSO_4_, 0.2 mM of dNTP mix, 1U of Platinum Taq DNA Polymerase High Fidelity (Invitrogen), and a mixture of the previously amplified fragments in a ratio of 2:1:2 (18 ng of the Left Flank: 9 ng of the fragment containing the P*groE* promoter, *lacI* and *cat* genes: 18 ng of the Right Flank). The PCR was carried out under the following conditions: 2 min of denaturation at 94 °C, followed by 10 cycles of 15 s at 94 °C, 40 s at 54 °C, and 2 min and 45 s at 68 °C. In the second reaction, 1.5 µL of the product from the first PCR was used as a template. The reaction was set up to a final volume of 25 µL, containing 1X of 10X High Fidelity Buffer, 3 mM of MgSO_4_, 0.2 mM of dNTP mix, 0.2 µM of primer A, 0.2 µM of primer F, and 1U of Platinum Taq DNA Polymerase High Fidelity. The PCR program was: 94 °C for 2 min, 25 cycles of 94 °C for 15 s, 60 °C for 30 s, 68 °C for 4 min and 30 s, and final extension at 68 °C for 7 min.

Purification of all PCR products was carried out using the IllustraTM GFXTM PCR DNA and Gel Band Purification Kit (GE Healthcare). Quantification of purified products was carried out using a Qubit^®^ 2.0 Fluorometer (InvitrogenTM).

### Cloning and Construction of the Vector pT-s*rfA*

The pGEM-T Easy Vector System (Promega) was utilized for cloning the integration cassette. E. coli DH5α cells were transformed with the TransformAid Bacterial Transformation Kit (Thermo Scientific). Ampicillin-resistant colonies were selected, and the recombinant plasmid was extracted using the GeneJET Plasmid Miniprep Kit (Thermo Scientific). PCR verification was performed using primers A and F (Table 1). The pGEM-T Easy vector carrying the integration cassette was designated as the pT-*srfA* integration vector.

### Induction of Competence and Transformation of *Bacillus subtilis* RI4914

Natural transformation of *B. subtilis* RI4914 was performed using the protocol developed by Vojcic et al. [21] with some modifications. First, to determine the time when *B. subtilis* RI4914 reached the state of competence (end of the exponential growth phase), the growth was monitored at the Starvation Medium 1 (SM1) and Starvation Medium 2 (SM2) by measuring the optical density at 600 nm over time. SM1 is composed of 2 g/L (NH_4_)_2_SO_4_, 14 g/L K_2_HPO_4_, 6 g/L KH_2_PO_4_, 0.8 g/L C_6_H_5_Na_3_O_7_.2H_2_O, 2 g/L yeast extract, 0.25 g/L hydrolyzed casein, 10 mL glucose (500 g/L), and 2.5 mL MgSO_4_.7H_2_O (80 g/L). Glucose and MgSO_4_.7H_2_O solutions were prepared separately, autoclaved, and added to the autoclaved medium. SM2 is composed of 2 g/L (NH_4_)_2_SO_4_, 14 g/L K_2_HPO_4_, 6 g/L KH_2_PO_4_, 0.8 g/L C_6_H_5_Na_3_O_7_.2H_2_O, 1 g/L yeast extract, 0.1 g/L hydrolyzed casein, 10 mL glucose (500 g/L), 10 mL MgSO_4_.7H_2_O (80 g/L), and 10 mL CaCl_2_ (100 mM), added to the medium after sterilization at 121 °C for 15 min. Histidine was added to SM2 at a final concentration of 200 µg/mL.

*B. subtilis* RI4914 cells were activated overnight in Petri dishes containing LB agar medium at 37 °C. A single colony was then transferred to glass tubes containing 5 mL of SM1 medium and incubated with agitation at 200 rpm and 37 °C for 12 h. The culture grown overnight was then transferred to 50 mL Erlenmeyer flasks containing 10 mL of SM1 medium, with the optical density at 600 nm (OD600 nm) adjusted to 0.5. To induce the competence, *B. subtilis* RI4914 was cultivated in a 50 mL Erlenmeyer flask containing 10 mL of SM1 at 37 °C and 200 rpm for 2 h and 15 min (reaching OD600 of 1.8). 10 mL of the culture was transferred to a 50 mL Erlenmeyer flask containing 10 mL of SM2. The flask was incubated under the same conditions for 2 h. To transform the competent cells, 500 µL of the competent cell suspension was transferred to a 2 mL microtube, and then 10 ng of the linearized pT-*srfA* vector was added. The tubes were incubated at 37 °C under shaking at 200 rpm for 30 min. Then, 300 µL of fresh LB medium was added to the tubes, and they were incubated again for 30 min. Subsequently, 200 µL of the cell suspension was spread-plated in LB Agar containing 5 µg/mL chloramphenicol. The plates were incubated at 37 °C for approximately 18 h.

Chloramphenicol-resistant colonies were then picked and checked by colony PCR. To confirm the integration of the cassette into the genomic DNA of *B. subtilis* RI4914 by homologous recombination, PCR was performed using the chromosomal DNA of transformant cells as template and primers A and F (Table 1). pT-*srfA* vector was used as the positive control, and the genomic DNA of the wild-type strains was used as the negative control.

### Genomic DNA Extraction

Genomic DNA extraction from the transformed cells *B. subtilis* LBBMA RI4914 was performed using the PowerSoil™ DNA Isolation Kit (MoBio, USA) according to the manufacturer’s instructions. The quality of the extracted DNA was analyzed on a 0.8% (w/v) agarose gel. DNA quantification was performed using a Qubit^®^ 2.0 Fluorometer (Invitrogen™).

### Genome Sequencing, Assembly, and Annotation

Approximately 4 µg of unsheared genomic DNA was processed using the Rapid Sequencing Kit (SQK-RAD004; Oxford Nanopore Technologies, UK). The prepared library was then sequenced on the GridION™ platform with a Spot-ON Mk1 flow cell (FLO-MIN 106 R9 version; Oxford Nanopore Technologies, UK) and an R9 Library Loading Bead Kit (EXP-LLB001; Oxford Nanopore Technologies, UK). Raw reads were obtained using MinKNOW software v3.5.6 during a 72-hour run and base-called with Albacore software v2.0.2. A subsample of the same DNA was also sequenced on the Ion Torrent PGM platform (Thermo Fisher Scientific, Waltham, MA, USA). The template library was prepared with the Ion Plus Fragment Library Kit and clonally amplified using the One Touch 2 System with the Ion PGM™ Template Hi-Q OT2 400 Kit. The amplified library was sequenced with the Ion PGM™ Hi-Q Sequencing 400 Kit on a 316™ Chip v2.

The de novo genome assembly was carried out using Flye version 2.9.3-b1797 [22] with raw ONT long reads (--nano-raw) and the scaffold method (--scaffold), applying five iterations of polishing (--iterations 5) based on an estimated genome size of 4 Mb (--genome-size 4 m). Subsequently, polishing with short-paired reads enhanced the accuracy of the assembled genome. Quality control of the short-read data was performed using FASTQC version 0.11.9 (https://github.com/s-andrews/FastQC). Adapter sequences were identified and removed using TrimGalore version 0.6.7 [23] with the auto-detection setting. The paired reads were then quality-trimmed and filtered for length using Trimmomatic version 0.39 [24] with the following parameters: HEADCROP:20, CROP:130, SLIDINGWINDOW:4:20, and MINLEN:100. Scaffold correction was achieved by mapping paired reads using the BWA-MEM algorithm in BWA version 0.7.17 [25], followed by five iterations of polishing with the Picard toolkit version 2.26.2 (https://github.com/broadinstitute/picard) and Pilon version 1.24 [26]. Genome completeness was assessed using BUSCO (Benchmarking Universal Single-Copy Orthologs) [27] in genome mode with the Bacillales order orthologous dataset (bacillales_odb10). Genome annotation was performed using the NCBI Prokaryotic Genome Annotation Pipeline (PGAP) [28].

### Production of Surfactin by Engineered and Wild-Type Strain

Wild and engineered colonies of *B. subtilis* were grown in 7 mL Tryptic Soy Broth (TSB; 17 g/L pancreatic digest of casein, 3 g/L peptic digest of soybean, 5 g/L NaCl, 2.5 g/L K_2_HPO_4_, 2.5 g/L glucose) at 30 °C and 200 rpm for 18 h. The seed cultures were inoculated into 125 mL Erlenmeyers flasks containing 30 mL of mineral medium comprised of 13.9 g/L K_2_HPO_4_, 2.7 g/L KH_2_PO_4_, 0.05 g/L yeast extract, 4.24 g/L NaNO_3_, 40 g/L glucose, and 50 mL of micronutrient solution (0.5 g/L EDTA, 3 g/L MgSO_4_.7H_2_O, 0.5 g/L MnSO_4_.4H_2_O, 1 g/L NaCl, 0.1 g/L CaCl_2_.2H_2_O, 0.1 g/L CoCl_2_.6H_2_O, 0.1 g/L ZnSO_4_.7H_2_O, 0.1 g/L FeSO_4_.7H_2_O, 0.01 g/L CuSO_4_.5H_2_O, 0.01 g/L Na_2_MoO_4_.2H_2_O, 0.01 g/L NaSeO_4_, 0.01 g/L Na_2_WoO_4_.2H_2_O, 0.02 g/L NiCl_2_) to achieve a starting OD_600_ of 0.05 (0 h). Both strains were cultivated in the absence and presence of Isopropyl β-D-1-thiogalactopyranoside (IPTG; 0.5 mmol/L), added at 0 h. The flasks were incubated at 30 °C and 200 rpm for 96 h. Cell densities, glucose consumption, and surfactin production were monitored over time by measuring optical density at 600 nm, glucose concentration by HPLC-RI, and oil spreading activity, respectively. Experiments were performed in triplicate with a randomized design.

To obtain the crude extract of surfactin at the end of incubation, cultures were centrifuged at 7,840 g for 15 min at room temperature. Surfactin was precipitated by adjusting the pH of the cell-free supernatant to 2.0 using HCl 6 mol/L and keeping it at 4 °C for approximately 20 h. The precipitate was collected by centrifugation (7,840 g; 15 min; room temperature) and dissolved in distilled water, adjusting the pH to 7.0 with NaOH 6 mol/L. The solution was frozen (-80 °C) and then lyophilized.

### Oil Spreading Test

The oil spreading test was performed according to the method described by Morikawa et al. [29] with some modifications. Briefly, 70 mL of distilled water was added to a Petri dish (150 × 20 mm), followed by the addition of 20 µL of crude oil to the water surface. Then, 10 µL of cell-free supernatant was dropped on the oil surface, and the diameter of the clear zone formed in the oil film was measured after 30 s. Youssef et al. [30] showed that the diameter of the clear zone resulting from the oil displacement activity of the surfactant has a linear relationship with the biosurfactant concentration.

### Glucose Consumption

Glucose concentration in the growth media was quantified using an LC-20AT liquid chromatographer equipped with a refractive index detector RID-20 A (Shimadzu). Aliquots of the culture broth were centrifuged at 10,000 *g* for 5 min, filtered through a 0.22 μm cellulose acetate membrane filter, and stored at -20 °C until analysis. The analysis was performed in an ion exchange column Aminex HPX-87 H (300 × 7.8 mm x 9 μm) with a mobile phase of H_2_SO_4_ 5 mM at 0.6 mL/min flow rate and 60 °C. The concentration of glucose was determined based on a standard curve (2.5 to 40 g/L glucose).

### Surfactin Quantification by Ultra-Performance Liquid Chromatography Coupled to Mass Spectrometry (UPLC-MS/MS)

Crude extracts of surfactin were solubilized in deionized water (Milli-Q) and acetonitrile (40:60 v/v) to a final concentration of 100 µg/mL. The solutions were centrifuged at 13,000 g for 10 min, and the supernatants were recovered. The surfactin standard curve was prepared by diluting a stock solution of surfactin (Sigma-Aldrich, 10 mg/mL) in deionized water (Milli-Q) and acetonitrile (40:60 v/v) to achieve the final concentrations of 100; 50; 25; 12.5; 6.25; 3.125; 1.5625; 0.78125; and 0.3906 µg/mL. The supernatants of the samples and standard solutions were filtered through a 0.22 μm cellulose acetate membrane filter directly into the vials and stored at -20 °C.

Surfactin quantification was carried out using a liquid phase chromatograph coupled to a triple quadrupole mass spectrometer (model 6430 - Agilent). 5 µL of each sample and standard solutions were injected into the UPLC liquid chromatograph (model 1200 Infinity series). Analysis was carried out using a Zorbax RRHD SB-CN column (150 × 3 mm x 1.8 μm) at a flow rate of 0.3 mL/min, 30 °C, and a mobile phase consisting of (A) deionized water-formic acid 0.1% (v/v) and (B) acetonitrile-formic acid 0.1% (v/v). The elution gradient was: 0–1.5 min, 45–55% (A-B); 1.5–8 min, 30–70%; 8–14 min, 0-100%; 14–16 min, 0-100%; 16–18 min, 45–55%. The mass spectrometer was operated at positive mode using the MRM (multiple reaction monitoring) method following the transitions listed in Table S2.

Three biological replications were performed for LC/MS analyses. The data were processed using the Skyline package version 21.1 (MacCoss Lab; University of Washington) as described by Vital et al. [31]. The transition between the precursor ion and product ion that presented the highest signal intensity was selected for the quantification of each molecule. Standard curves were constructed for each detected precursor ion, based on the relative abundance of each molecule in the standard. The concentration of each surfactin molecule in the samples was determined based on its respective standard curve. The final surfactin concentration corresponds to the sum of the concentrations of all detected surfactin molecules.

### Statistical Analysis

Results are expressed as mean ± standard deviation. Unless otherwise stated, all experiments were performed in triplicate using three independent biological replicates. Statistical analyses were performed using ANOVA followed by Bonferroni’s multiple comparison test at a significance level of 5% using GraphPad Prism version 5.04.

### Genome Sequence Accession Numbers

The complete genome sequence of *Bacillus subtilis* RI4914 I*srfA* chromosome has been deposited in GenBank under accession number CP154865. The plasmid sequence associated with the engineered construct is available under accession number CP154866.

## Results

### Construction of the Integration Cassette by Overlap Extension PCR and Cloning

The extraction of genomic DNA from *B. subtilis* LBBMA RI4914 was carried out successfully and its integrity was confirmed by electrophoretic analysis (Figure S2). Left and Right Flank fragments that constitute the arms of the cassette and that were homologous to the *B. subtilis* chromosome were obtained by PCR from the chromosomal DNA and contain 845 bp and 773 bp, respectively (Figure S2). According to Yan et al. [32], the minimum length of the homologous region necessary for the integration of a linear DNA to occur in *B. subtilis* is approximately 500 bp. The third fragment of the cassette, including the P*grac* promoter region, *lacI*, and *cat* genes, was successfully amplified from the pHT43 vector (2,775 pb; Figure S2).

The presence of a band corresponding to the expected size of the integration cassette (4,365 bp) confirmed the successful assembly of the fragments by overlap extension PCR (Figure S2). The Overlap Extension PCR technique offers greater speed and flexibility for the construction of recombinant DNA molecules, when compared to laborious conventional techniques dependent on restriction and ligation enzymes [33, 34].

pT-*srfA* vector was obtained after cloning the integration cassette into the pGEM-T Easy vector. Recombinant clones were successfully obtained, and the presence of the integration cassette in the plasmid pT-*srfA* was confirmed by colony PCR (4,365 pb; Figure S3).

### Transformation of *B. subtilis* RI4914 and Obtaining of the Engineered Strain *B. subtilis* RI4914 I*srfA*

**Fig. 3:**
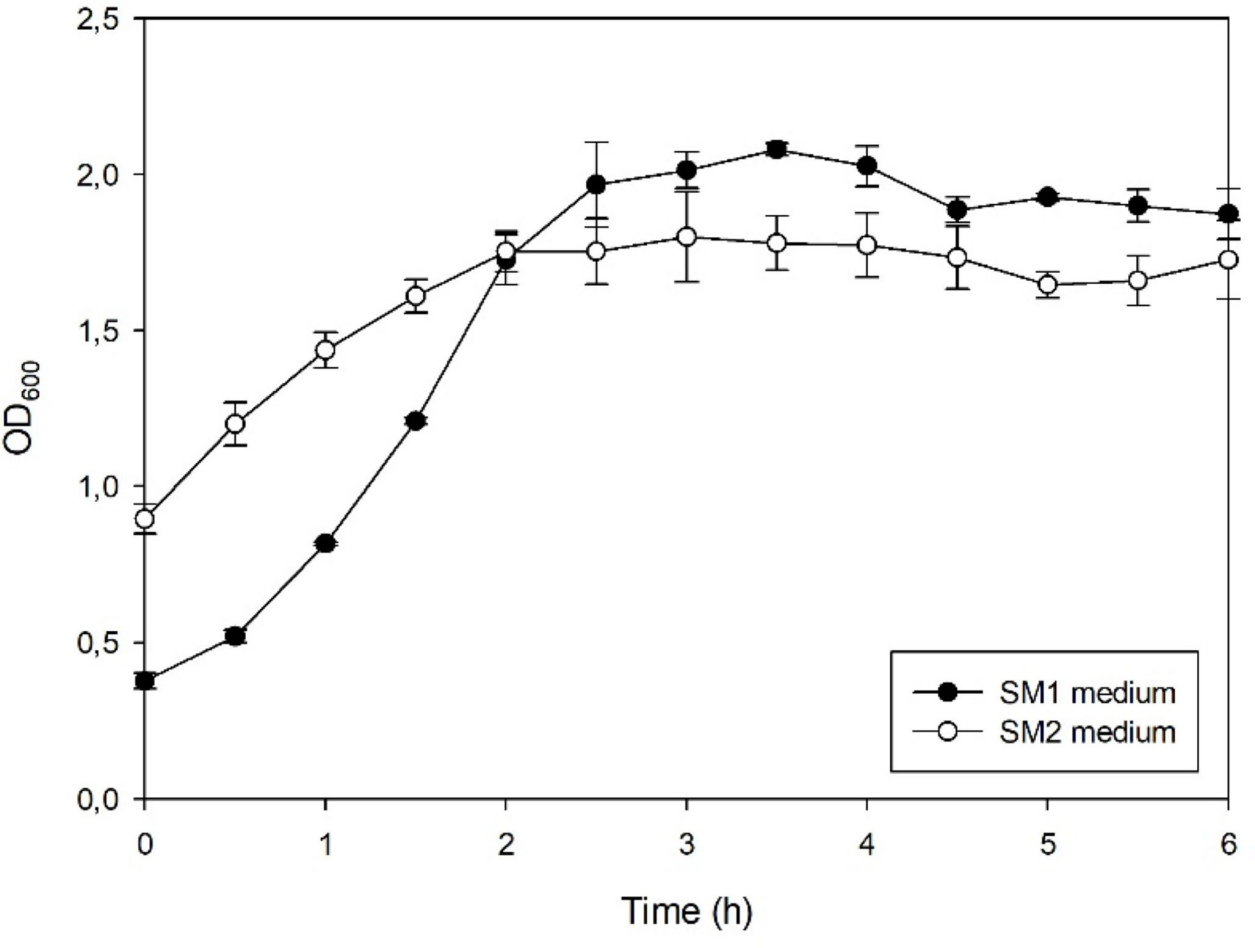
Growth curve of *B. subtilis* RI4914 in SM1 and SM2 media. Results are expressed as mean ± standard deviation of values obtained for three repetitions

*B. subtilis* develops its natural competence at the end of the exponential growth phase [35]. In *B. subtilis* RI4914, this physiological state was reached after cultivation for 2 h and 15 min in the SM1 medium, followed by growth in the SM2 medium for 2 h (Fig. 3).

Chloramphenicol-resistant colonies were picked from the plate and submitted to colony PCR to confirm the transformation. Integration of the cassette into the genome was confirmed by PCR, comparing the amplicons obtained from chromosomal DNA of wild type (WT) and engineered strains. The presence of a fragment with a size compatible with the integration cassette (4,365 bp) was confirmed in all transforming strains (Figure S4). The amplified band of the WT was smaller than the others, corresponding to the size of the original sequence of the *srfA* operon. This result indicated the occurrence of double cross-over recombination and integration of the cassette into the chromosome of *B. subtilis* LBBMA RI4914.

One of the colonies was chosen to continue the experiments and was named *B. subtilis* LBBMA RI4914 I*srfA*. The integration of the cassette between *hxIR* and *srfAA* and the exchange of the native promoter of the *srfA* operon with the P*grac* promoter were confirmed by analyzing the sequence of the genome of the engineered strain, deposited at NCBI under the accession number CP154865 (Figure S1). The genome size was 4,101,244 bp with a G + C content of 66.82%.

### Production of Surfactin by Modified and Wild-Type Strains

**Fig. 4:**
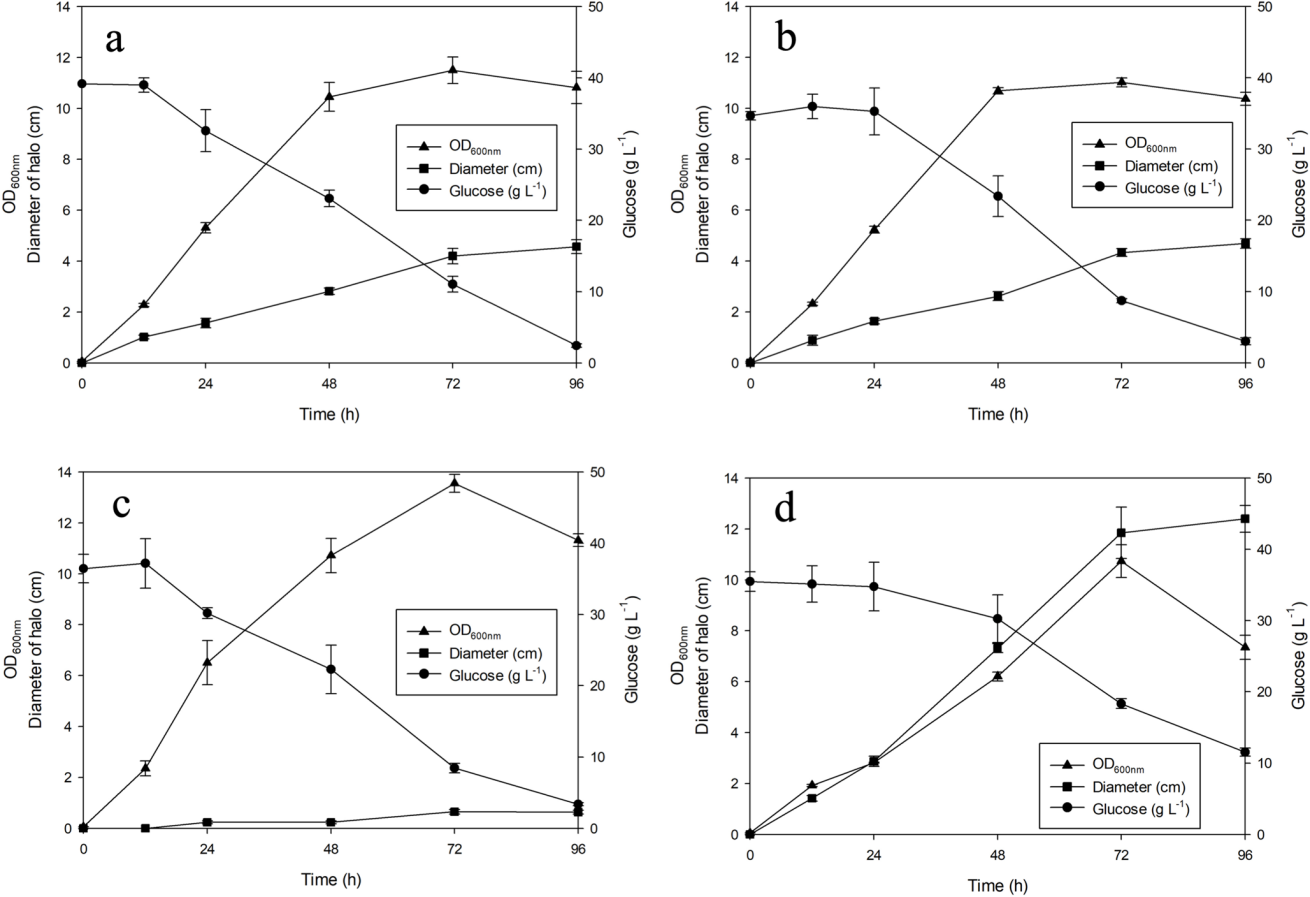
Kinetics of growth, halo diameter (oil spreading test) and consumption of glucose of the *B. subtilis* RI4914 WT cultured in mineral medium without (**a**) and with IPTG (**b**) and *B. subtilis* RI4914 I*srfA* cultured in mineral medium without (**c**) and with IPTG (**d**). Results are expressed as mean ± standard deviation of values obtained for three repetitions

Figure 4 represents the kinetics of growth, surfactin production, and consumption of glucose of the *B. subtilis* RI4914 WT and *B. subtilis* RI4914 I*srfA* strains grown in the presence and absence of IPTG. Growth, biosurfactant production, and glucose consumption of *B. subtilis* RI4914 WT were not affected by IPTG (Fig. 4A, B). *B. subtilis* RI4914 WT achieves the maximum cell growth at 48 h and then enters the stationary phase. Surfactin production, measured by the oil spreading test, starts at the early exponential phase and remains in the same pattern throughout cell growth, regardless of the presence of IPTG (Fig. 4A, B).

On the other hand, the presence of IPTG strongly influenced surfactin production by *B. subtilis* RI4914 I*srfA*. In the absence of the inducer, the diameter of the halo varied from 0 to 0.6 cm during the period of incubation (Fig. 4C). In contrast, a halo of 12 cm was observed at 96 h when *B. subtilis* RI4914 I*srfA* was grown in medium containing IPTG (Fig. 4D). Glucose consumption and biomass production were lower in the presence of IPTG. The presence of IPTG from the beginning of incubation may have redirected cellular metabolism toward surfactin synthesis, which is consistent with an increased expression of the srfA operon expected under control of the inducible Pgrac promoter. However, because no direct transcriptional analyses were performed, this interpretation remains indirect and is inferred from surfactin production behavior. In other words, a large part of the energy and precursors necessary for cell growth was redirected to the production of surfactin and, as a result, the production of biomass was lower, and surfactin production was higher (Fig. 4C, D).

## Discussion

The lower biomass accumulation observed in the engineered strain under IPTG induction may indicate a redistribution of cellular resources associated with increased surfactin synthesis. A similar trend was reported by Liu et al. [10], who observed lower biomass production in a high surfactin-yielding strain. This pattern may reflect a shift in carbon and energy allocation toward metabolite production rather than biomass formation.

**Fig. 5:**
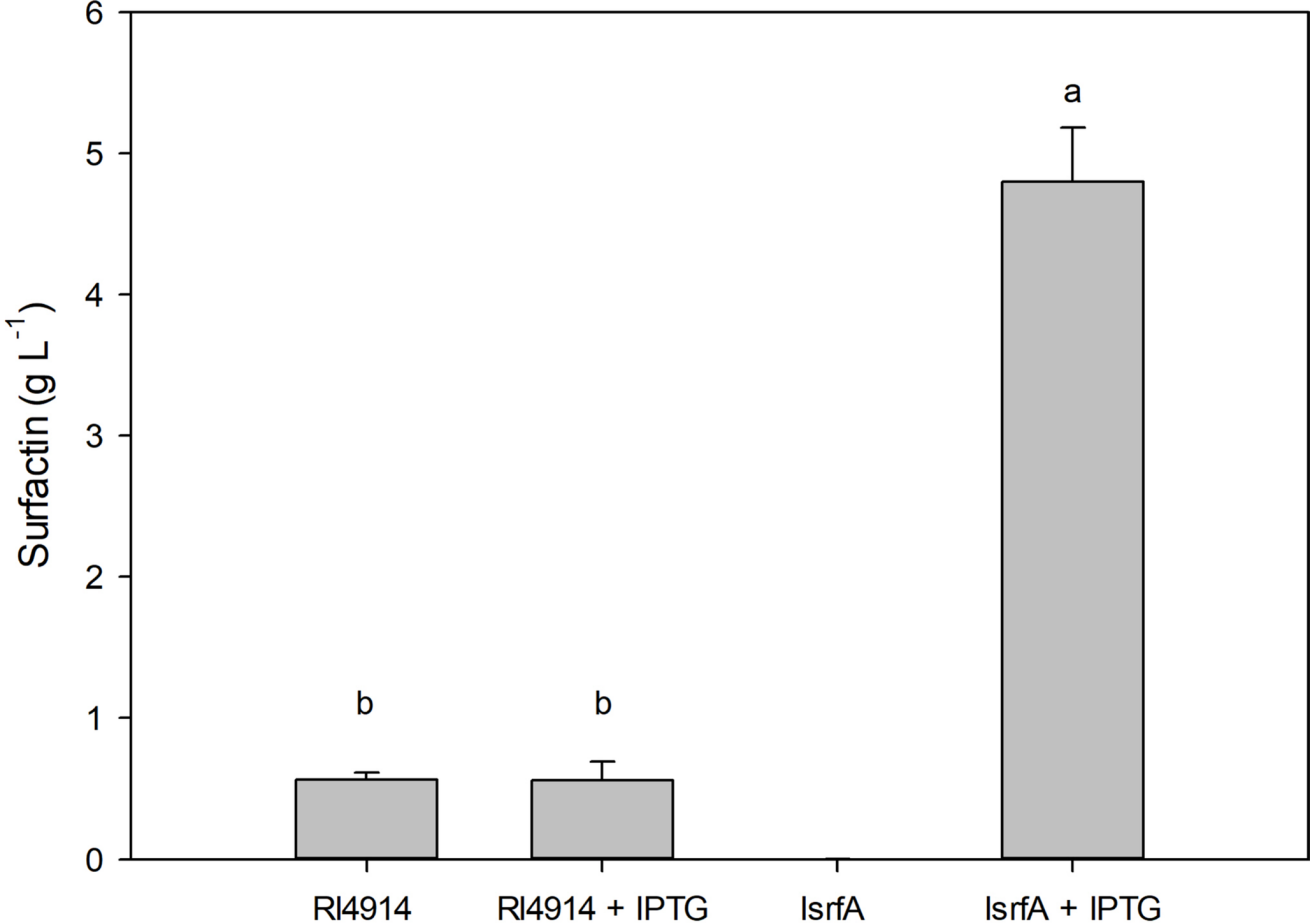
Surfactin titer of the wild and modified strains cultured in mineral medium with and without IPTG for 96 h. Results are expressed as mean ± standard deviation of values obtained for three repetitions. Means with the same letter do not differ statistically by Bonferroni test at 5% of probability

Wu et al. [36] systematically enhanced surfactin biosynthesis in *B. subtilis* 168 by reintroducing the *sfp* gene, deleting genes associated with biofilm formation and nonribosomal peptide synthesis, and overexpressing genes linked to surfactin export and cellular resistance. This multifaceted approach led to a remarkable 20.3-fold increase in surfactin titer, reaching concentrations of 8.5 g/L. Additionally, optimizing branched-chain fatty acid biosynthesis was critical in supplying sufficient precursors for surfactin synthesis. In contrast, the promoter engineering strategy employed in our study, while less complex, reached 4.8 g/L and yielded an 8.4-fold increase in surfactin production under IPTG induction (Fig. 5; Table 2). This highlights the effectiveness of targeted promoter engineering strategies in improving surfactin production. In contrast to more complex metabolic engineering approaches involving multiple gene deletions or extensive pathway rewiring [37–39], the strategy employed in this study relies on a single promoter replacement event. The substantial increase in surfactin production observed in *Bacillus subtilis* RI4914 demonstrates that minimal genetic modifications can already produce significant improvements in biosurfactant yield. Our results, therefore, suggest that promoter engineering offers a practical and scalable alternative to extensive metabolic modifications, potentially simplifying the pathway to industrial-scale biosurfactant production.

**Table 2:** Comparison of production metrics of *B. subtilis* RI4914 and *B. subtilis* RI4914 in the presence (+) and absence (-) of IPTG

Strain	Titer (g/L)	Productivity (g/L/h)	Carbon source yield (g/g)
RI4914 WT - IPTG	0,57	0,006	0,015
RI4914 WT + IPTG	0,56	0,007	0,017
RI4914 IsrfA - IPTG	0,002	0,000	0,000
RI4914 IsrfA + IPTG	4,8	0,050	0,171

While several studies have focused on complex metabolic engineering strategies involving multiple genetic modifications to improve surfactin production [40], the present work demonstrates that a single regulatory modification (replacement of the native P*srfA* promoter with the inducible P*grac* promoter) can substantially increase surfactin production in *Bacillus subtilis* RI4914.

Comparing the production metrics of fermentation in shake flasks, the production of surfactin reached a productivity of 0.05 g/L/h and a carbon source yield of 0.17 g/g glucose for RI4914 I*srfA*, which is 8.4-fold and 11-fold higher than that achieved by the WT, respectively (Table 2). The carbon source yield obtained for *B. subtilis* RI4914 I*srfA* grown in a shake flask was higher than that observed by Jiao et al. [14] for *B. subtilis* THY-7/Pg3-srfA in a 5 L fermentor (0.14 g surfactin/g sucrose). These data demonstrate the great fermentative performance of *B. subtilis* RI4914 I*srfA* and indicate that this strain has potential for industrial application.

Given the importance of promoters in gene expression, many studies have focused on replacing promoters in order to obtain greater expression of genes of interest to increase the production of bacterial metabolites [14, 16, 17]. Some studies reported that replacing the P*srfA* promoter with strong constitutive promoters was not satisfactory for the production of surfactin [14, 41, 42]. Some authors argue that strong promoters under the control of an inducer are the most advantageous ones for replacing the P*srfA* promoter [14, 16]. Our results showed that introduction of the IPTG-inducible Pgrac promoter was associated with a strong increase in surfactin production. In this context, our findings contribute to the development of improved *Bacillus subtilis* strains for surfactin production and reinforce the potential of regulatory engineering as a practical alternative to more complex metabolic engineering strategies. To our knowledge, this is the first study demonstrating promoter replacement of the *srfA* operon in the environmental strain *Bacillus subtilis* RI4914, resulting in a substantial increase in surfactin production.

Various approaches have been investigated to increase surfactin production, including optimization of culture conditions, metabolic engineering of precursor pathways, and regulatory modifications of the *srfA* operon [40, 43, 44]. Although metabolic pathway engineering can lead to substantial improvements in production, it typically requires multiple genetic modifications and extensive optimization of complex regulatory networks [39], making it time-consuming and labor-intensive. In contrast, promoter replacement represents a more straightforward strategy that can be implemented more rapidly, requiring fewer genetic interventions while still enabling effective transcriptional control of biosynthetic genes and enhancement of product formation.

Replacement of the P*srfA* promoter with P*grac* in *B. subtilis* RI4914 I*srfA* significantly increased surfactin titer, productivity, and yield. As a result, a high surfactin-producing strain with potential application in industry was obtained. This work constitutes the first step towards enabling industrial production and commercial use of surfactin by *B. subtilis* RI4914 I*srfA*. Further improvements in surfactin synthesis and reduction of production cost can be achieved through optimization of fermentation parameters, downstream processing, and utilization of cheap substrates (e.g., agro-industrial residues), in order to make the process economically viable.

## Conclusions

In this study, replacement of the native PsrfA promoter with the IPTG-inducible Pgrac promoter successfully generated an engineered *Bacillus subtilis* RI4914 strain with substantially improved surfactin production. The modified strain reached a surfactin titer of 4.8 g/L and showed significant increases in productivity and substrate yield compared with the wild-type strain. These findings demonstrate that promoter replacement can serve as an effective and relatively simple strategy to improve surfactin production without extensive metabolic engineering. Although the enhanced promoter activity was inferred indirectly from surfactin production behavior, the engineered strain presents promising potential for future optimization and industrial biosurfactant applications.

## Supplementary Information

Below is the link to the electronic supplementary material.

Supplementary Material 1

Supplementary Material 2

Supplementary Material 3

Supplementary Material 4

Supplementary Material 5

Supplementary Material 6

## Data Availability

Data available on request from the authors.
