# Supplementary material for: Promoter Engineering of the Surfactin Operon Enhances Surfactin Production in the Environmental Strain Bacillus subtilis RI4914
Source: Curr Microbiol. 2026 Jun 30;83(8):460. doi: 10.1007/s00284-026-05037-3 (PMC13319662; doi:10.1007/s00284-026-05037-3)
Supplement: Supplementary file 1 — Supplementary Material 1 [file 284_2026_5037_MOESM1_ESM.docx]

ACAAAAATGTCATGAAAGAATCGTTGTAAGACGCTCTACGCAAGGGTGTCTTTTTTTTGCCTGTTTTTCGGTTTTTGCGCGGTACACATAGTCATGTAAAGATTGTAAATTGCATTCAGCAATAAAAAAAGATTGAACGCAGCAGTTTGGTTTGAAAATTTTTATTTTTCTGTAAATAATGTTTAGTGGAAATGATTGCGGCATCCCGCAAAAATATCCCTGTAAATAAACTGGAATATTTCGGCATCCCGCATGAAACTTTTCACCCATTTTTCGGTGATAAAAACATTTTTTTCATTTAAAGTGAACGGTAGAAAGATAAAAAATATTGAAAACAATGAATAAATAGCCGAAATTGGTTTCTTATTAGGATGGGGTCTTGCGGTCTTTATCCGCTTGCGTTAAACGCCGCAATACTGACTGACGGCAGCCTGCTTTAATAGCGGCCATCTGTTTTTTGATTGGAAGCACTGCTTTTTAAGTACTTTGGGCTATTTCGTCTGTTAGTTCATAAGAATTAAAAGCTGATATGGATAAGAAAGAGAAAATGCGTTGCGTAAAAAGTACAGTCGGCATTATCTCATATTATAAAAGCCAGTCATTAGGCCTATCTGACAATTCCTGAATAGAGTTCATAAACAATCCTGCATGATAACCATCACAAACAGAATGATGTACCTGTAAAGATAGCGGTAAATATATTGAATTACCTTTATTAATGAATTTTCCTGCTGTAATAATGGGTAGAAGGTAATTACTATTATTATTGATATTTAAGTTAAACCCAGTAAATGAAGTCCATGGAATAATAGAAAGAGAAAAAGCATTTTCAGGTATAGGTGTTTTGGGAAGCAATTTCCCCGAACCATTATATTTCTCTACATCAGAAAGGTATAAATCATAAAACTCTTTGAAGTCATTCTTTACAGGAGTCCAAATACCAGAGAATGTTTTAGATACACCATCAAAAATTGTATAAAGTGGCTCTAACTTATCCCAATAACCTAACTCTCCGTCGCTATTGTAACCAGTTCTAAAAGCTGTATTTGAGTTTATCACCCTTGTCACTAAGAAAATAAATGCAGGGTAAAATTTATATCCTTCTTGTTTTATGTTTCGGTATAAAACACTAATATCAATTTCTGTGGTTATACTAAAAGTCGTTTGTTGGTTCAAATAATGATTAAATATCTCTTTTCTCTTCCAATTGTCTAAATCAATTTTATTAAAGTTCATTTGATATGCCTCCTAAATTTTTATCTAAAGTGGATTTAGGAGGCTTACTTGTCTGCTTTCTTCATTAGAATCAATCCTTTTTAAAAGTCAATATTACTGTAACATAAATATATATTTTAAAAATATCCCACTTTATCCAATTTTCGTTTGTTGAACTAATGGGTGCTTTAGTTGAAGAATAAAAGACCACATTAAAAAATGTGGTCTTTTGTGTTTTTTTAAAGGATTTGAGCGTAGCGAAAAATCCTTTTCTTTCTTATCTTGATAATAAGGGTAACTATTGCCGTCGTCCATTCCGACAGCATCGCCAGTCACTATGGCGTGCTGCTAGCGTCATTCGCCATTCAGGCTGCGCAACTGTTGGGAAGGGCGATCGGTGCGGGCCTCTTCGCTATTACGCCAGCTGGCGAAAGGGGGATGTGCTGCAAGGCGATTAAGTTGGGTAACGCCAGGGTTTTCCCAGTCACGACGTTGTAAAACGACGGCCAGTGAATTCGAGCTCAGGCCTTAACTCACATTAATTGCGTTGCGCTCACTGCCCGCTTTCCAGTCGGGAAACCTGTCGTGCCAGCTGCATTAATGAATCGGCCAACGCGCGGGGAGAGGCGGTTTGCGTATTGGGCGCCAGGGTGGTTTTTCTTTTCACCAGTGAGACGGGCAACAGCTGATTGCCCTTCACCGCCTGACCCTGAGAGAGTTGCAGCAAGCGGTCCACGCTGGTTTGCCCCAGCAGGCGAAAATCCTGTTTGATGGTGGTTAACGGCGGGATATAACATGAGCTGTCTTCGGTATCGTCGTATCCCACTACCGAGATATCCGCACCAACGCGCAGCCCGGACTCGGTAATGGCGCGCATTGCGCCCAGCGCCATCTGATCGTTGGCAACCAGCATCGCAGTGGGAACGATGCCCTCATTCAGCATTTGCATGGTTTGTTGAAAACCGGACATGGCACTCCAGTCGCCTTCCCGTTCCGCTATCGGCTGAATTTGATTGCGAGTGAGATATTTATGCCAGCCAGCCAGACGCAGACGCGCCGAGACAGAACTTAATGGGCCCGCTAACAGCGCGATTTGCTGGTGACCCAATGCGACCAGATGCTCCACGCCCAGTCGCGTACCGTCTTCATGGGAGAAAATAATACTGTTGATGGGTGTCTGGTCAGGGACATCAAGAAATAACGCCGGAACATTAGTGCAGGCAGCTTCCACAGCAATGGCATCCTGGTCATCCAGCGGATAGTTAATGATCAGCCCACTGACGCGTTGCGCGAGAAGATTGTGCACCGCCGTTTTACAGGCTTCGACGCCGCTTCGTTCTACCATCGACACCACCACGCTGGCACCCAGTTGATCGGCGCGAGATTTAATCGCCGCGACAATTTGCGACGGCGCGTGCAGGGCCAGACTGGAGGTGGCAACGCCAATCAGCAACGACTGTTTGCCCGCCAGTTGTTGTGCCACGCGGTTGGGAATGTAATTCAGCTCCGCCATCGCCGCTTCCACTTTTTCCCGCGTTTTCGCAGAAACGTGGCTGGCCTGGTTCACCACGCGGGAAACGGTCTGATAAGAGACACCGGCATACTCTGCGACATCGTATAACGTTACTGGTTTCATCAAAATCGTCTCCCTCCGTTTGAATATTTGATTGATCGTAACCAGATGAAGCACTCTTTCCACTATCCCTACAGTGTTATGGCTTGAACAATCACGAAACAATAATTGGTACGTACGATCTTTCAGCCGACTCAAACATCAAATCTTACAAATGTAGTCTTTGAAAGTATTACATATGTAAGATTTAAATGCAACCGTTTTTTCGGAAGGAAATGATGACCTCGTTTCCACCGGAATTAGCTTGGTACCAGCTATTGTAACATAATCGGTACGGGGGTGAAAAAGCTAACGGAAAAGGGAGCGGAAAAGAATGATGTAAGCGTGAAAAATTTTTTATCTTATCACTTGAAATTGGAAGGGAGATTCTTTATTATAAGAATTGTGGAATTGTGAGCGGATAACAATTCCCAATTAAAGGAGGAAGGATCA**ATG**GAAATAACTTTTTACCCTTTAACGGATGCACAAAAACGAATTTGGTACACAGAAAAATTTTATCCTCACACGAGCATTTCAAATCTTGCAGGGATTGG

Figure S1. Genomic DNA (5’–3’) of *Bacillus subtilis* RI4914 IsrfA in front of srfA operon. Part of the genome sequence was obtained from NCBI (accession number CP154865) from 212570 bp to 215950 bp. The rho-independent terminator sequence of *hxIR* is indicated by yellow characters. The *cat* gene is emphasized in orange and the *lacI* gene is indicated in green. The regions -10 and -35 of the Pgrac promoter are emphasized in red. *lacO* operator is indicated in blue. The start of the *srfAA* ORF is indicated by a dashed underline with the initial ATG highlighted in pink.
