# Supplementary material for: Promoter Engineering of the Surfactin Operon Enhances Surfactin Production in the Environmental Strain Bacillus subtilis RI4914
Source: Curr Microbiol. 2026 Jun 30;83(8):460. doi: 10.1007/s00284-026-05037-3 (PMC13319662; doi:10.1007/s00284-026-05037-3)
Supplement: Supplementary file 2 — Supplementary Material 2 [file 284_2026_5037_MOESM2_ESM.docx]

Table S1. PCR reactions for amplification of the fragments that make up the integration cassette

| Component | Final concentration | | |
| --- | --- | --- | --- |
|  | Product AB* | Product CD* | Product EF* |
| Template | 10 ng | 40 ng | 10 ng |
| Primer A | 0.3 μM | - | - |
| Primer B | 0.3 μM | - | - |
| Primer C | - | 0.3 μM | - |
| Primer D | - | 0.3 μM | - |
| Primer E | - | - | 0.3 μM |
| Primer F | - | - | 0.3 μM |
| 10X Pfx Amplification Buffer | 1X | 1X | 1X |
| MgSO_4_ | 1 mM | 1 mM | 1 mM |
| dNTP Mix | 300 μM | 300 μM | 300 μM |
| Platinum® Pfx DNA Polymerase | 1U | 1U | 1U |
| H_2_O | Para 50μL | Para 50μL | Para 50μL |

* Product AB = *Left Flank*; Product CD = fragment conteinig P*grac* promoter, *lacI* and *cat* genes; Product EF = *Right Flank*.
