# Supplementary material for: Promoter Engineering of the Surfactin Operon Enhances Surfactin Production in the Environmental Strain Bacillus subtilis RI4914
Source: Curr Microbiol. 2026 Jun 30;83(8):460. doi: 10.1007/s00284-026-05037-3 (PMC13319662; doi:10.1007/s00284-026-05037-3)
Supplement: Supplementary file 3 — Supplementary Material 3 [file 284_2026_5037_MOESM3_ESM.docx]

Table S2. MRM transitions, retention times, and collision energy used for analysis of surfactin molecules by UPLC-MS/MS

| Precursor Ion  [M + H]^+^ (*m/z*) | Product Ion  (*m/z*) | Retention time  (min) | Collision energy  (eV) |
| --- | --- | --- | --- |
| 1050.7 | 699.4 | 5.38 | 35 |
|  | 685.3 | 5.38 | 35 |
|  | 610.4 | 5.38 | 35 |
|  | 596.4 | 5.38 | 35 |
|  | 441.3 | 5.38 | 35 |
| 1036.7 | 685.3 | 5.25 | 35 |
|  | 582.4 | 5.25 | 35 |
|  | 596.4 | 5.25 | 35 |
|  | 677.4 | 5.25 | 35 |
|  | 699.4 | 5.25 | 35 |
| 1022.7 | 582.4 | 5.03 | 35 |
|  | 664.4 | 5.03 | 35 |
|  | 685.4 | 5.03 | 35 |
|  | 596.4 | 5.18 | 35 |
|  | 671.4 | 5.18 | 35 |
| 1008.7 | 441.3 | 4.89 | 35 |
|  | 568.4 | 4.89 | 35 |
|  | 685.4 | 4.89 | 35 |
|  | 582.4 | 5.04 | 35 |
|  | 671.4 | 5.04 | 35 |
| 994.6 | 441.3 | 4.75 | 35 |
|  | 554.4 | 4.75 | 35 |
|  | 685.3 | 4.75 | 35 |
