# Supplementary material for: Promoter Engineering of the Surfactin Operon Enhances Surfactin Production in the Environmental Strain Bacillus subtilis RI4914
Source: Curr Microbiol. 2026 Jun 30;83(8):460. doi: 10.1007/s00284-026-05037-3 (PMC13319662; doi:10.1007/s00284-026-05037-3)
Supplement: Supplementary file 4 — Supplementary Material 4 [file 284_2026_5037_MOESM4_ESM.docx]

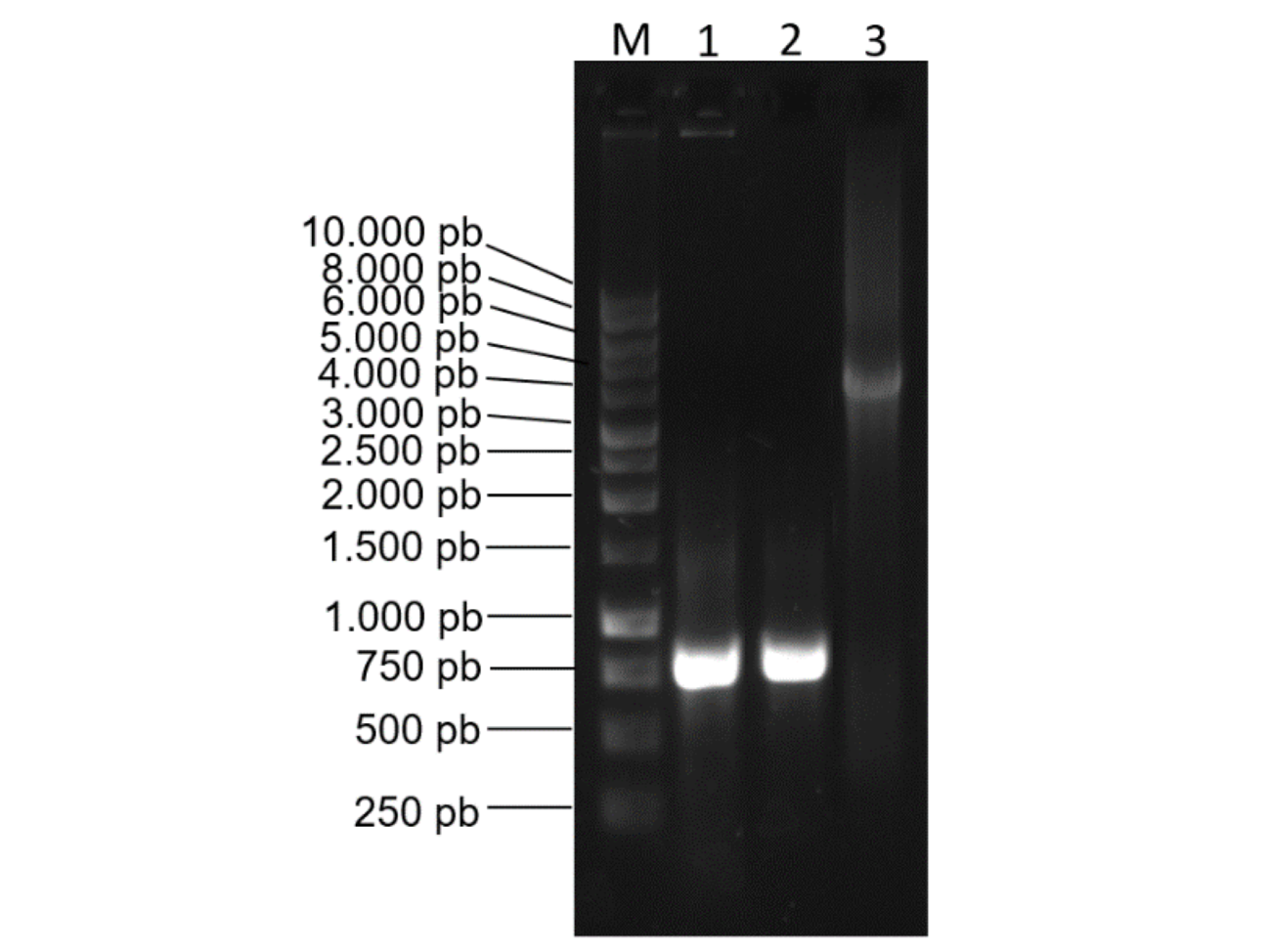


Figure S3. Confirmation of cloning of the integration cassette into the pGEM-T Easy vector. M: 1 Kb DNA Ladder (Promega). 1 and 2: Amplicon of the Right Flank fragment (799 bp) from the colony and positive control. 3: Amplicon corresponding to the integration cassette (4,365 bp).
