# Supplementary material for: Promoter Engineering of the Surfactin Operon Enhances Surfactin Production in the Environmental Strain Bacillus subtilis RI4914
Source: Curr Microbiol. 2026 Jun 30;83(8):460. doi: 10.1007/s00284-026-05037-3 (PMC13319662; doi:10.1007/s00284-026-05037-3)
Supplement: Supplementary file 5 — Supplementary Material 5 [file 284_2026_5037_MOESM5_ESM.docx]

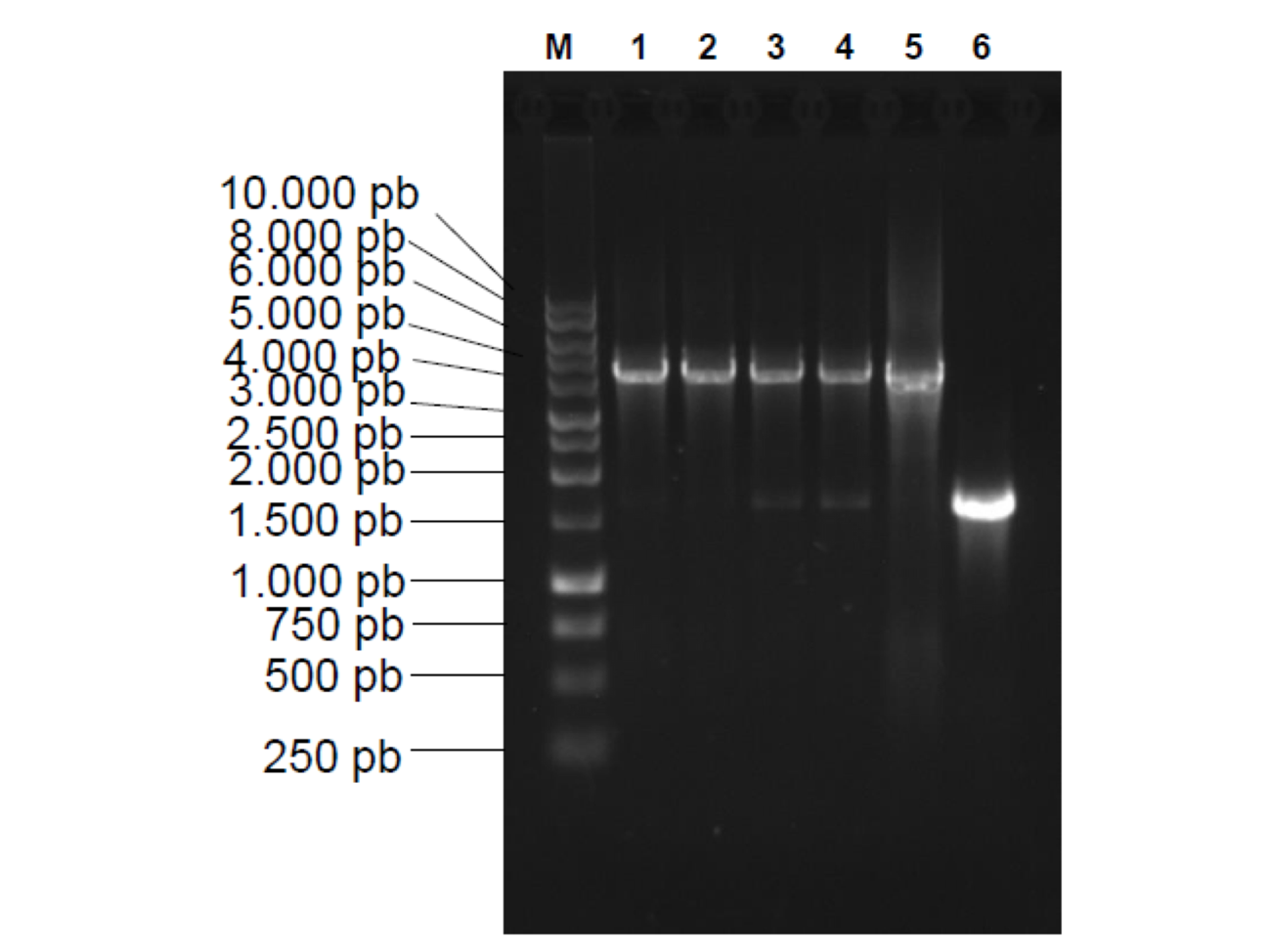


Figure S4. Confirmation of the integration of the cassette into the genome of B. subtilis RI4914 by homologous recombination. M: 1 Kb DNA Ladder size marker (Promega); 1 to 4: Amplicons of the integration cassette of the B. subtilis RI4914 transformants strains (4,365 bp); 5: positive control (pT-srfA); 6: Amplicon of the srfA region of B. subtilis RI4914 WT (1,659 bp).
