# Supplementary material for: Promoter Engineering of the Surfactin Operon Enhances Surfactin Production in the Environmental Strain Bacillus subtilis RI4914
Source: Curr Microbiol. 2026 Jun 30;83(8):460. doi: 10.1007/s00284-026-05037-3 (PMC13319662; doi:10.1007/s00284-026-05037-3)
Supplement: Supplementary file 6 — Supplementary Material 6 [file 284_2026_5037_MOESM6_ESM.docx]

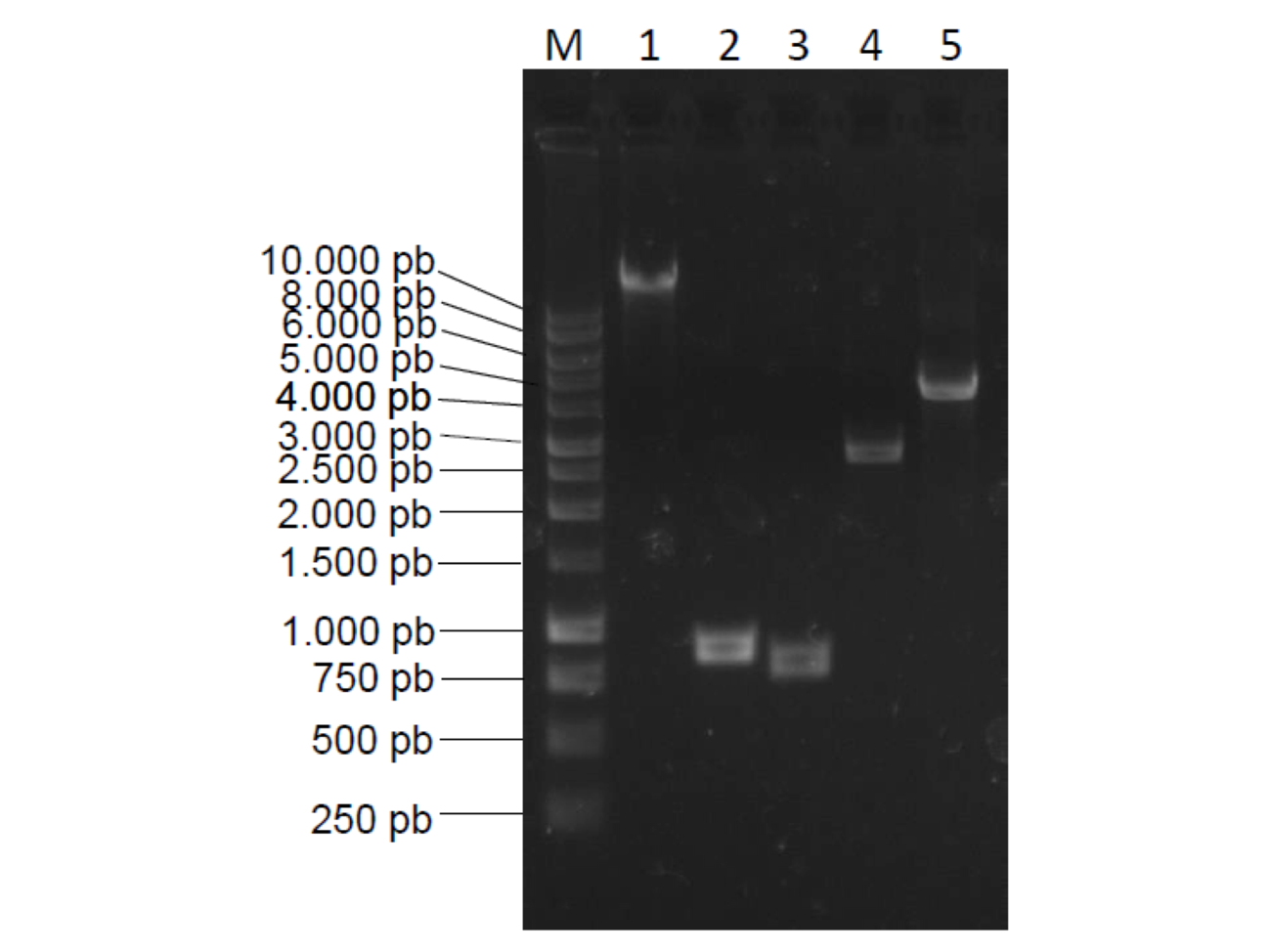


Figure S2. Confirmation of obtaining fragments and integration cassette by PCR. M: 1Kb DNA Ladder (Promega). 1: Genomic DNA of B. subtilis RI4914. 2: Amplicon corresponding to the Left Flank fragment (871 bp); 3: Amplicon corresponding to the Right Flank fragment (799 bp); 4: Amplicon corresponding to the fragment containing the Pgrac promoter, lacI, and cat genes (2,755 bp); 5: Amplicon corresponding to the integration cassette (4,365 bp).
